# Personalized prediction of mortality in patients with acute ischemic stroke using explainable artificial intelligence

**DOI:** 10.1186/s40001-024-01940-2

**Published:** 2024-06-20

**Authors:** Lingyu Xu, Chenyu Li, Jiaqi Zhang, Chen Guan, Long Zhao, Xuefei Shen, Ningxin Zhang, Tianyang Li, Chengyu Yang, Bin Zhou, Quandong Bu, Yan Xu

**Affiliations:** 1 Department of Nephrology, The Affiliated Hospital of Qingdao University, 16 Jiangsu Road, Qingdao, 266003 China; 2 Division of Nephrology, Medizinische Klinik Und Poliklinik IV, Klinikum der Universität, Munich, Germany; 3 Yidu Central Hospital of Weifang, Weifang, China

**Keywords:** Acute ischemic stroke, Artificial intelligence, Acute kidney disease, Machine learning, Mortality

## Abstract

**Background:**

Research into the acute kidney disease (AKD) after acute ischemic stroke (AIS) is rare, and how clinical features influence its prognosis remain unknown. We aim to employ interpretable machine learning (ML) models to study AIS and clarify its decision-making process in identifying the risk of mortality.

**Methods:**

We conducted a retrospective cohort study involving AIS patients from January 2020 to June 2021. Patient data were randomly divided into training and test sets. Eight ML algorithms were employed to construct predictive models for mortality. The performance of the best model was evaluated using various metrics. Furthermore, we created an artificial intelligence (AI)-driven web application that leveraged the top ten most crucial features for mortality prediction.

**Results:**

The study cohort consisted of 1633 AIS patients, among whom 257 (15.74%) developed subacute AKD, 173 (10.59%) experienced AKI recovery, and 65 (3.98%) met criteria for both AKI and AKD. The mortality rate stood at 4.84%. The LightGBM model displayed superior performance, boasting an AUROC of 0.96 for mortality prediction. The top five features linked to mortality were ACEI/ARE, renal function trajectories, neutrophil count, diuretics, and serum creatinine. Moreover, we designed a web application using the LightGBM model to estimate mortality risk.

**Conclusions:**

Complete renal function trajectories, including AKI and AKD, are vital for fitting mortality in AIS patients. An interpretable ML model effectively clarified its decision-making process for identifying AIS patients at risk of mortality. The AI-driven web application has the potential to contribute to the development of personalized early mortality prevention.

**Supplementary Information:**

The online version contains supplementary material available at 10.1186/s40001-024-01940-2.

## Keypoints

- What was known: Acute kidney disease (AKD) defines patients with acute kidney injury (AKI) or subacute loss of kidney function lasting for more than 7 days, which links well AKI to subsequent chronic kidney disease (CKD). Little is known about the risk and prognosis of AKD in acute ischemic stroke (AIS) patients.
- This study adds: Renal function trajectories, including both AKI and AKD, play a crucial role in predicting mortality in AIS patients. The LightGBM model elucidates decision processes by providing explanations at both global and local levels. The AI web application aids in reducing mortality rates and helps physicians make informed treatment decisions.
- Potential impact: Adding AKD as a definition for renal failure lasting > 7 days up to 90 days is of clinical importance in addition to the existing definitions for AKI and CKD. Research activities and clinical practice should also focus on AKD, which is far more accurate to predict prognosis especially mortality.

**Supplementary Information:**

The online version contains supplementary material available at 10.1186/s40001-024-01940-2.

## Background

The global impact of stroke is substantial, ranking second in mortality and third in disability, with an estimated annual cost exceeding US$891 billion worldwide [1, 2]. Notably, ischemic strokes constituted over 60% of all stroke events [3]. Renal impairment is a critical adverse complication in AIS patients, often induced by factors such as mechanical thrombectomy, which increases the risk of mortality [4–6]. Existing research has primarily focused on AKI and CKD, with a scarcity of reports addressing the renal function trajectory during the 7–90 days following kidney injury [7, 8].

AKI and CKD do not represent distinct clinical syndromes but rather frequently present as a disease continuum [9]. No consensus exists for defining criteria to evaluate kidney recovery after AKI [10]. The 2012 Kidney Disease Improving Global Outcomes (KDIGO) guideline first introduced the term ‘Acute Kidney Diseases and Disorders’, defining it as abnormalities in kidney function and/or structure lasting less than 3 months, which includes AKI [11]. The 2017 Acute Disease Quality Initiative (ADQI) workgroup defines acute kidney disease (AKD) as acute or subacute damage and/or loss of kidney function persisting for 7 to 90 days following an AKI-triggering event [12]. Although the diagnostic criteria for AKD differ between the two guidelines, both stress the importance of considering AKD as a condition of equal significance to AKI.

Artificial intelligence (AI) is at the forefront of digital medicine [13]. Machine learning (ML), a fundamental branch of AI, excels in deciphering complex nonlinear associations among multidimensional features [14]. It has been extensively applied in the realm of healthcare, spanning areas such as medical diagnostics and the prediction of disease risks [15, 16]. Numerous studies employ ML models to predict mortality risk in patients with conditions such as heart failure, surgical interventions, and sepsis [17–19]. These studies predominantly utilize decision tree-based algorithms, which handle nonlinear features more effectively and mitigate overfitting compared to traditional regression models. In addition, ML significantly enhances outcome interpretability by elucidating influential variables, complex internal operations, and learned decision-making paths. SHapley Additive exPlanations (SHAP), a prominent interpretive method, quantify the marginal contribution of each feature upon integration into a ‘black-box’ model, providing explanations at both global and local levels [20, 21]. Its strength lies in precisely measuring the impact’s degree and direction that each feature exerts on the model’s output. In assessing mortality risk for AIS patients, research primarily focuses on those in intensive care unit (ICU) [22, 23], which creates a gap in prognostic evaluations for non-ICU AIS patients. Studies involving non-ICU AIS patients face challenges related to imbalanced data distribution, with a mortality rate of less than 5%, and this imbalance remains unaddressed [24]. Importantly, there is a dearth of research dedicated to predicting the impact of AKD on the mortality of AIS patients.

Hence, this study aimed to achieve the following objectives: (1) evaluate the incidence of AKI, AKD, and mortality among AIS patients; (2) assess mortality risk using various ML algorithms and identify the most optimal model; (3) utilize SHAP analysis to elucidate the contributions of individual features to the outcome and unveil the underlying decision-making process; (4) compare the predictive capabilities of using AKD independently or in combination with AKI for predicting mortality; (5) develop a user-friendly online prediction tool for estimating the probability of mortality in AIS patients.

## Materials and methods

### Study design

This retrospective cohort study involved 1633 patients diagnosed with AIS between January 2020 and June 2021. All patients were randomly assigned to a test set comprising 15% of samples not seen during model development; this set was used to assess the final model’s performance. An 85% sample subset was designated as the training set for model building. During the training phase, we employed a grid search with tenfold cross-validation to fine-tune model hyperparameters and prevent overfitting [25].

Patients diagnosed with AIS were included according to the International Classification of Diseases version 10 (ICD-10). Individuals meeting any of the following criteria were excluded: (1) age < 18 years; (2) hospitalization duration < 24 h; (3) hospital-acquired or traumatic brain injury with concurrent stroke, or comorbid intracranial tumor, transient ischemic attack, or other intracranial disorders; (4) concurrent Stage 5 CKD, undergoing renal replacement therapy, or having undergone kidney transplant; and (5) patients with incomplete data recording.

### Data collection

Clinical information was extracted using natural language processing and parsing methods applied to structured data within the electronic health record. Data pertaining to demographic characteristics, medical history, and comorbidities were collected upon admission. Medication records were compiled during hospitalization, with particular attention to instances where these medications were administered before the onset of kidney injury. Comprehensive blood counts, coagulation markers, blood chemistry analyses, and urine tests were conducted within 1 week of admission. Initially, we included 104 readily available features based on expert clinical opinions and literature reviews. Following the removal of features with a missing proportion greater than 15%, we retained 86 features for building the prediction models.

### Outcome definitions

The study investigated AKI and AKD as short-term outcomes, and mortality as a long-term outcome. AKI was defined in accordance with the 2012 KDIGO criteria, signifying either a rise in serum creatinine (Scr) greater than 0.3 mg/dL from baseline within 48 h or an increase to 1.5 times the baseline value within 7 days [11]. As stipulated by the 2017 ADQI guidelines, AKD was characterized by the acute or subacute impairment and/or loss of kidney function occurring within 7 to 90 days following an AKI event [12]. Based on the diagnostic criteria for AKI and AKD, patients exhibited three distinct renal function trajectories following kidney injury: (1) AKI recovery, indicating that Scr returned to baseline value within 7 days; (2) subacute AKD, denoting a slow increase in Scr levels lasting more than 7 days (AKD without AKI); and (3) AKD with AKI, representing the persistence of stage 1 or greater AKI for ≥ 7 days after an AKI initiating event (AKI progressing to AKD). The final classification encompassed four categories: (1) no kidney disease (NKD), (2) AKI recovery, (3) subacute AKD, and (4) AKD with AKI. Mortality was defined by the vital status for survival or death at the last follow-up. Clinical features, incorporating renal function trajectories, were incorporated to develop a risk prediction model, with mortality as the binary endpoint, to evaluate mortality risk in AIS patients.

The baseline Scr level was defined as the initial Scr measurement obtained upon hospital admission. The timing of AKI and AKD diagnosis was determined when patients initially met the respective diagnostic criteria. Each patient underwent a minimum of three Scr tests, which included two tests during their hospitalization and one at their first follow-up appointment. If elevated Scr levels did not return to baseline, additional tests were performed weekly during hospitalization or at the subsequent follow-up. The estimated glomerular filtration rate (eGFR) was calculated using the Chronic Kidney Disease Epidemiology Collaboration (CKD-EPI) creatinine formula [26].

### Model development and interpretation

Data were trained on the following eight ML models: (1) light gradient boosting machine (LightGBM), (2) GBM, (3) random forest (RF), (4) K-nearest neighbors (KNN), (5) multi-layer perceptron (MLP), (6) naive Bayes (NB), (7) support vector machine (SVM), and (8) logistic regression (LR). LightGBM and GBM are gradient-based learning frameworks that employ decision trees and boosting. LightGBM, in comparison to GBM, shortens training times and reduces memory usage by partitioning data using histograms [27]. RF constructs individual decision trees using random subsets of the training data and combines their results through majority voting for classification [28]. KNN is a frequently used supervised learning algorithm that conducts classification or regression based on feature similarity among neighboring data points [29]. MLP relies on the stacking of multiple layers of neurons, employing layer-wise propagation and nonlinear activation functions to learn and represent intricate data relationships [30]. NB is rooted in Bayes’ theorem and performs classification by calculating the posterior probabilities of different categories under given feature conditions [31]. SVM is a supervised learning algorithm that makes predictions by identifying the optimal separating hyperplane [32]. LR is a linear model that predicts probabilities based on the logistic function [33]. All models using the same dataset and applying consistent imputation and scaling techniques.

SHAP was used to interpret the results of the top-performing model. Features with positive SHAP values enhance the output, with larger numerical values indicating more significant contributions [34]. SHAP summary plots offer visualizations of essential feature rankings and the overarching relationships and directions concerning features and outcomes. SHAP force and decision plots offer an intuitive visualization of how distinct features influence an individual prediction.

### Data balancing

In our study, there exists an imbalance, as the mortality rate is approximately 5%. To address this imbalance, we utilized a weight rebalancing technique to adjust the weights of both the majority and minority classes [35]. Solely the training dataset underwent balancing. The test datasets remained unaltered to evaluate model performance using representative data. The scikit-learn Python library includes a built-in parameter called “class weight” or “weights” for LR, RF, LightGBM, SVM, and KNN. The model automatically assigns a weight to each class that is inversely proportional to its frequency. The balanced weight for each class is calculated using the equation: Class weight = total number of samples/(number of classes × class sample size). The class weight for mortality was 10.34, while the class weight for non-mortality was 0.53 when the “balanced” option was used. In the case of the NB classifier, we established a prior probability of 0.5 for each class to achieve group balance. In future work, we plan to adjust class weights in the MLP classifier by modifying the loss function’s weights.

### AI-driven web application

A web-based calculator for predicting mortality among AIS patients was developed using the “Streamlit” application (https://share.streamlit.io/) to implement the optimal model [36]. To enhance the user-friendliness of the web calculator, this study introduced two panels: one for inputting model parameters and obtaining mortality probability, and another for providing a model introduction.

### Statistical analysis

Features with missing values exceeding 15% were omitted from the dataset. Multiple imputation techniques were then applied to estimate the missing data. Utilizing LR to compute the required sample size with mortality as the outcome, we ascertained that a minimum of 801 patients is essential to achieve a statistical power of 90% for the detection of an effect size of 0.10 at a two-sided significance level (α) of 0.05. Normally distributed continuous features are reported as the median ± standard deviation (SD) and were compared using independent *t* test. For non-normally distributed features, we present them as the median (interquartile range) and utilized the Mann–Whitney *U* test for comparisons. Categorical features were characterized in terms of percentages and underwent comparison through the Pearson’s Chi-squared test. We evaluated the models’ predictive performance using a variety of metrics, including the area under the receiver operating characteristic curve (AUROC), precision, recall, accuracy, F1 score, Brier score loss (BSL), Matthew’s correlation coefficient, and decision curve analysis (DCA). The AUROC and F1 score were utilized to identify the optimal model. A significance level of less than 0.05 (two-tailed) was utilized. Our analysis was conducted using the Python programming language (Python Software Foundation, version 3.9.13) within the integrated development environment Visual Studio Code 1.81.1.

## Results

### Study cohort

**Fig. 1:**
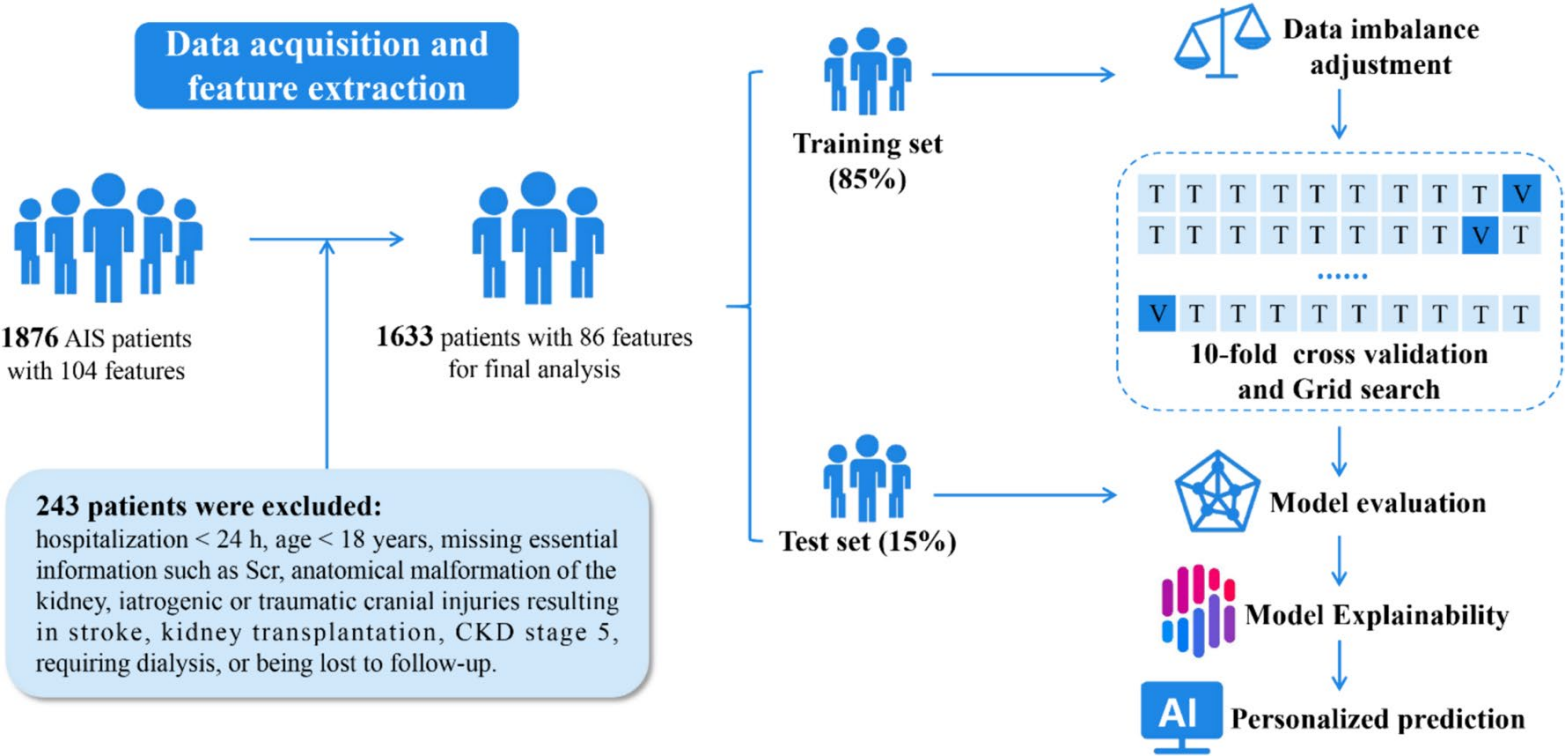
Architectural diagram of study

A retrospective review of medical records was conducted for 1876 AIS patients from January 2020 to June 2021, with 1633 were eligible for further analysis (Fig. 1). Table 1 presents the baseline characteristics of the study population, and Table S1 stratifies the same cohort based on mortality. The incidence rates of AKI, AKD, and mortality were 14.57% (238/1633), 19.72% (322/1633), and 4.84% (79/1633), respectively. From the perspective of renal function trajectories, a total of 495 patients (30.31%) developed acute/subacute kidney dysfunction (meeting AKI and/or AKD criteria), comprising 257 patients (15.74%) with subacute AKD, 173 patients (10.59%) who experienced recovery from AKI, and 65 patients (3.98%) meeting both AKI and AKD criteria. Increased mortality rates were noted in elderly individuals (mean age: 73 vs. 68 years), those experiencing fever (15.19% vs. 8.04%), and patients with AKD coupled with AKI (31.65% vs. 13.92% in subacute AKD, 25.32% in AKI recovery, and 29.11% in NKD patients).

**Table 1:** Baseline characteristics of inpatients [mean ± SD; *n* (%)]

Features	Total (*n* = 1633)	NKD (*n* = 1138)	Acute/subacute renal impairment (*n* = 495)			
			AKI recovery (*n* = 173)	Subacute AKD (*n* = 257)	AKD with AKI (*n* = 65)	*P*-value
Demographics						
Age (years)	68.37 ± 13.38	68.24 ± 13.46	67.06 ± 13.98	69.30 ± 11.99	70.45 ± 15.23	0.55
Male (%)	991 (60.69)	691 (60.72)	101 (58.38)	165 (64.20)	34 (52.31)	1
Smokers (%)	559 (34.23)	395 (34.71)	57 (32.95)	88 (34.24)	19 (29.23)	0.57
Drinkers (%)	442 (27.07)	307 (26.98)	47 (27.17)	77 (29.96)	11 (16.92)	0.95
Surgical history (%)	579 (35.46)	390 (34.27)	60 (34.68)	109 (42.41)	20 (30.77)	0.14
Blood transfusion (%)	167 (10.23)	101 (8.88)	30 (17.34)	26 (10.12)	10 (15.38)	**0.01**
BMI (kg/m^2^)	23.99 ± 3.22	23.95 ± 3.15	24.20 ± 3.38	24.00 ± 3.42	24.13 ± 3.32	0.42
SBP (mmHg)	142.79 ± 23.71	142.33 ± 22.71	142.18 ± 25.69	144.73 ± 25.24	144.77 ± 28.79	0.24
DBP (mmHg)	81.34 ± 13.99	81.18 ± 13.55	81.29 ± 15.36	82.12 ± 14.21	81.14 ± 16.88	0.49
Fever (%)	137 (8.39)	95 (8.35)	13 (7.51)	21 (8.17)	8 (12.31)	1
Respiratory rate (bpm)						
< 12	3 (0.18)	1 (0.09)	1 (0.58)	1 (0.39)	0 (0.00)	0.13
12–20	1371 (83.96)	970 (85.24)	142 (82.08)	211 (82.10)	48 (73.85)	
> 20	259 (15.86)	167 (14.67)	30 (17.34)	45 (17.51)	17 (26.15)	
Heart rate (bpm)						
< 60	135 (8.27)	93 (8.17)	20 (11.56)	20 (7.78)	2 (3.08)	0.18
60–100	1376 (84.26)	965 (84.80)	140 (80.92)	211 (82.10)	60 (92.31)	
> 100	122 (7.47)	80 (7.03)	13 (7.51)	26 (10.12)	3 (4.62)	
Laboratory tests						
RBC (× 10^12^/L)	4.20 ± 0.76	4.24 ± 0.72	4.06 ± 0.93	4.21 ± 0.75	3.96 ± 0.81	**0.01**
WBC (× 10^9^/L)	8.27 ± 4.30	7.96 ± 3.96	9.54 ± 4.11	8.14 ± 4.36	10.95 ± 7.54	** < 0.01**
Neutrophil count (× 10^9^/L)	5.89 ± 3.62	5.56 ± 3.20	7.44 ± 3.99	5.67 ± 3.92	8.53 ± 5.59	** < 0.01**
Hemoglobin (g/L)	126.30 ± 23.92	127.12 ± 23.17	122.21 ± 27.72	126.99 ± 23.85	120.05 ± 24.85	**0.04**
Platelet (× 10^9^/L)	217.02 ± 80.16	220.04 ± 79.67	207.49 ± 91.85	212.74 ± 69.98	206.52 ± 90.69	**0.02**
MCV (fL)	89.88 ± 6.19	89.65 ± 6.21	90.36 ± 6.68	90.12 ± 5.26	91.64 ± 7.59	**0.02**
Hematocrit (%)	37.66 ± 6.86	37.83 ± 6.58	36.55 ± 8.11	37.86 ± 7.01	36.91 ± 7.36	0.14
MCHC (g/L)	334.36 ± 14.96	334.83 ± 14.91	331.73 ± 17.11	334.46 ± 13.32	332.72 ± 15.22	0.05
MCH (pg)	30.06 ± 2.43	30.03 ± 2.49	29.97 ± 2.56	30.14 ± 2.06	30.45 ± 2.40	0.47
PT (s)	11.44 ± 3.07	11.29 ± 2.79	11.94 ± 3.21	11.63 ± 3.94	12.06 ± 3.31	** < 0.01**
PTA (%)	110.89 ± 32.64	112.42 ± 32.75	103.35 ± 28.00	109.94 ± 32.15	107.84 ± 40.63	** < 0.01**
Fibrinogen (g/L)	3.62 ± 1.10	3.62 ± 1.10	3.70 ± 1.15	3.53 ± 1.10	3.80 ± 1.01	1
Scr (μmol/L)	102.74 ± 99.40	96.87 ± 73.86	146.73 ± 205.03	102.36 ± 91.89	90.01 ± 47.58	** < 0.01**
Bassline eGFR (ml/min/1.73 m^2^)	72.43 ± 25.28	72.92 ± 23.81	72.50 ± 34.59	70.12 ± 23.44	72.84 ± 28.08	0.24
BUN (mmol/L)	7.01 ± 4.87	6.94 ± 5.15	7.18 ± 3.65	7.35 ± 4.72	6.45 ± 2.70	0.38
Uric acid (μmol/L)	289.96 ± 121.17	285.10 ± 111.32	301.23 ± 161.68	299.71 ± 114.52	306.46 ± 173.41	**0.01**
ALT (U/L)	37.52 ± 173.35	29.84 ± 52.17	57.22 ± 171.09	54.22 ± 392.56	53.42 ± 139.13	**0.01**
AST (U/L)	35.22 ± 158.95	25.86 ± 29.57	60.74 ± 185.51	48.78 ± 343.63	77.48 ± 241.00	** < 0.01**
GGT (U/L)	43.37 ± 83.36	41.18 ± 74.95	51.24 ± 87.30	48.08 ± 115.50	42.20 ± 56.68	0.11
ADA (U/L)	12.51 ± 7.18	12.42 ± 7.43	13.26 ± 7.16	12.60 ± 6.58	11.74 ± 4.37	0.44
LDH (U/L)	211.61 ± 160.31	197.80 ± 109.05	282.95 ± 273.07	203.42 ± 119.54	295.85 ± 402.37	** < 0.01**
ALP (U/L)	80.32 ± 54.03	79.20 ± 47.59	83.40 ± 57.54	83.06 ± 77.08	80.84 ± 38.36	0.2
Total bilirubin (μmol/L)	17.76 ± 17.93	17.11 ± 15.02	21.92 ± 34.25	17.80 ± 14.22	17.94 ± 13.27	**0.03**
Total protein (g/L)	62.77 ± 7.65	62.81 ± 7.34	62.59 ± 8.66	63.18 ± 8.12	60.95 ± 8.11	0.76
Albumin (g/L)	34.71 ± 5.86	34.92 ± 5.67	33.71 ± 6.21	35.00 ± 6.31	32.61 ± 5.77	**0.03**
HDLC (mmol/L)	1.18 ± 0.36	1.18 ± 0.35	1.14 ± 0.49	1.20 ± 0.32	1.16 ± 0.36	0.86
LDLC (mmol/L)	2.55 ± 1.04	2.56 ± 1.02	2.46 ± 1.05	2.58 ± 1.07	2.44 ± 1.29	0.52
Total cholesterol (mmol/L)	4.48 ± 1.56	4.48 ± 1.44	4.47 ± 2.21	4.49 ± 1.42	4.44 ± 2.10	0.95
Triglycerides (mmol/L)	1.38 ± 1.15	1.36 ± 1.04	1.57 ± 1.85	1.35 ± 0.84	1.27 ± 1.55	0.36
Lipoprotein a (mg/L)	353.00 ± 380.48	326.11 ± 309.98	547.75 ± 717.17	336.54 ± 303.92	370.43 ± 340.58	** < 0.01**
Blood glucose (mmol/L)	7.18 ± 3.52	6.90 ± 3.18	8.77 ± 4.78	7.11 ± 3.47	8.03 ± 4.12	** < 0.01**
Sodium (mmol/L)	140.44 ± 5.02	140.52 ± 4.82	139.51 ± 6.40	140.85 ± 4.38	139.95 ± 6.40	0.36
Calcium (mmol/L)	2.14 ± 0.18	2.15 ± 0.18	2.16 ± 0.20	2.09 ± 0.18	2.12 ± 0.21	**0.01**
Potassium (mmol/L)	4.03 ± 0.55	4.05 ± 0.53	4.05 ± 0.58	4.02 ± 0.64	3.82 ± 0.59	0.14
Magnesium (mmol/L)	0.89 ± 0.12	0.91 ± 0.12	0.85 ± 0.10	0.89 ± 0.12	0.84 ± 0.10	** < 0.01**
Chloride (mmol/L)	103.32 ± 5.64	103.60 ± 5.43	103.10 ± 6.23	102.65 ± 5.86	101.73 ± 6.30	** < 0.01**
Phosphorus (mmol/L)	1.04 ± 0.30	1.05 ± 0.30	1.06 ± 0.26	0.97 ± 0.29	1.00 ± 0.24	**0.01**
Anion gap (mmol/L)	12.26 ± 3.26	12.41 ± 3.19	11.55 ± 3.21	12.23 ± 3.50	11.45 ± 3.40	** < 0.01**
Urinalysis						
pH	6.01 ± 0.60	5.98 ± 0.60	6.14 ± 0.64	5.99 ± 0.57	6.20 ± 0.66	** < 0.01**
Specific gravity	1.02 ± 0.01	1.02 ± 0.01	1.01 ± 0.01	1.02 ± 0.01	1.01 ± 0.01	** < 0.01**
Protein	501 (30.68)	334 (29.35)	65 (37.57)	83 (32.30)	19 (29.23)	0.09
Glucose	935 (57.26)	610 (53.60)	93 (53.76)	189 (73.54)	43 (66.15)	** < 0.01**
Hematuria	1304 (79.85)	854 (75.04)	149 (86.13)	242 (94.16)	59 (90.77)	** < 0.01**
Positive fecal occult blood, *n* (%)	122 (7.47)	77 (6.77)	10 (5.78)	33 (12.84)	2 (3.08)	0.12
Comorbidities, *n* (%)						
Cerebral hemorrhage	110 (6.74)	81 (7.12)	9 (5.20)	16 (6.23)	4 (6.15)	0.41
Epilepsy	31 (1.90)	20 (1.76)	3 (1.73)	8 (3.11)	0 (0.00)	0.66
Cerebral aneurysm	37 (2.27)	30 (2.64)	4 (2.31)	3 (1.17)	0 (0.00)	0.18
Shock	16 (0.98)	10 (0.88)	1 (0.58)	4 (1.56)	1 (1.54)	0.72
Diabetes mellitus	565 (34.60)	379 (33.30)	65 (37.57)	101 (39.30)	20 (30.77)	0.11
Hypertension	1103 (67.54)	765 (67.22)	115 (66.47)	183 (71.21)	40 (61.54)	0.72
CHD	580 (35.52)	391 (34.36)	71 (41.04)	95 (36.96)	23 (35.38)	0.15
Heart failure	271 (16.60)	183 (16.08)	23 (13.29)	56 (21.79)	9 (13.85)	0.44
Myocardial infarction	92 (5.63)	57 (5.01)	12 (6.94)	18 (7.00)	5 (7.69)	0.12
Cardiac arrhythmia	359 (21.98)	236 (20.74)	46 (26.59)	58 (22.57)	19 (29.23)	0.08
Urinary tract infection	50 (3.06)	29 (2.55)	7 (4.05)	11 (4.28)	3 (4.62)	0.09
CKD	115 (7.04)	66 (5.80)	27 (15.61)	13 (5.06)	9 (13.85)	** < 0.01**
COPD	44 (2.69)	27 (2.37)	8 (4.62)	8 (3.11)	1 (1.54)	0.29
Pulmonary infection	415 (25.41)	270 (23.73)	34 (19.65)	96 (37.35)	15 (23.08)	**0.02**
Gastrointestinal bleeding	31 (1.90)	23 (2.02)	0 (0.00)	6 (2.33)	2 (3.08)	0.72
Medications, *n* (%)						
ACEI/ARB	881 (53.95)	598 (52.55)	91 (52.60)	147 (57.20)	45 (69.23)	0.1
CCB	776 (47.52)	527 (46.31)	86 (49.71)	127 (49.42)	36 (55.38)	0.15
β-Blocker	559 (34.23)	352 (30.93)	84 (48.55)	85 (33.07)	38 (58.46)	** < 0.01**
Diuretics	1081 (66.20)	714 (62.74)	138 (79.77)	171 (66.54)	58 (89.23)	** < 0.01**
Proton pump inhibitors	1073 (65.71)	716 (62.92)	132 (76.30)	169 (65.76)	56 (86.15)	** < 0.01**
Statins	1119 (68.52)	780 (68.54)	119 (68.79)	175 (68.09)	45 (69.23)	1
Antibiotics	987 (60.44)	649 (57.03)	132 (76.30)	149 (57.98)	57 (87.69)	** < 0.01**
NSAIDs	204 (12.49)	148 (13.01)	17 (9.83)	34 (13.23)	5 (7.69)	0.38
Metformin	232 (14.21)	171 (15.03)	19 (10.98)	36 (14.01)	6 (9.23)	0.17
Antithrombotic drugs	1442 (88.30)	1013 (89.02)	153 (88.44)	222 (86.38)	54 (83.08)	0.2
Adrenergic drugs	291 (17.82)	205 (18.01)	26 (15.03)	49 (19.07)	11 (16.92)	0.81
Cardiac glycosides	253 (15.49)	150 (13.18)	21 (12.14)	59 (22.96)	23 (35.38)	** < 0.01**
Outcome						
Mortality, *n* (%)	79 (4.84)	23 (2.02)	20 (11.56)	11 (4.28)	25 (38.46)	** < 0.01**
Renal function grade, *n* (%)						
0	1138 (69.69)	1138 (100.00)	0 (0.00)	0 (0.00)	0 (0.00)	** < 0.01**
1	359 (21.98)	0 (0.00)	140 (80.92)	189 (73.54)	30 (46.15)	
2	82 (5.02)	0 (0.00)	20 (11.56)	47 (18.29)	15 (23.08)	
3	54 (3.31)	0 (0.00)	13 (7.51)	21 (8.17)	20 (30.77)	
Length of stay (days)	23.02 ± 12.51	23.52 ± 11.68	23.23 ± 19.29	21.25 ± 9.02	20.75 ± 14.70	**0.01**

SD: Standard deviation; NKD: No kidney disease; AKI: Acute kidney injury; AKD: Acute kidney disease; BMI: Body mass index; SBP: Systolic blood pressure; DBP: Diastolic blood pressure; RBC: Red blood cell; WBC: White blood cell; MCV: Mean corpuscular volume; MCHC: Mean corpuscular hemoglobin concentration; MCH: Mean corpuscular hemoglobin; PT: Prothrombin time; PTA: Prothrombin activity; Scr: Serum creatinine; eGFR: Estimated glomerular filtration rate; BUN: Blood urea nitrogen; ALT: Alanine transaminase; AST: Aspartate transaminase; GGT: Gamma-glutamyl transferase; ADA: Adenosine deaminase; LDH: Lactate dehydrogenase; ALP: Alkaline phosphatase; HDLC: High-density lipoprotein cholesterol; LDLC: Low-density lipoprotein cholesterol; CHD: Coronary heart disease; CKD: Chronic kidney disease; COPD: Chronic obstructive pulmonary disease; ACEI/ARB: Angiotensin-converting enzyme inhibitor/Angiotensin receptor blocker; CCB: Calcium channel blocker; NSAIDs: Non-steroidal anti-inflammatory drugs

### Model performance

A comprehensive set of 86 features served as predictors for mortality and were integrated into the ML models. Among all ML models, the LightGBM model displayed the best performance, with an AUROC of 0.96 and an F1 score of 0.47 (Fig. S1, Table S1, and Table S2). After data balancing, the model showed no significant difference in AUROC and accuracy, but it achieved a better balance between precision and recall (Table 2 and Table S3). When the model incorporated only the top 10 features, the AUROC remained high at 0.93, while maintaining a balance between precision and recall. Consequently, the LightGBM model was utilized in later stages for result interpretation and the development of an AI-driven web application. DCA revealed that the LightGBM model possessed high clinical utility (Fig. S2). Additional information concerning various performance metrics, such as accuracy, BSL, and Matthews correlation coefficient, is available in Table 2 and Table S2.

**Table 2:** Performance of LightGBM model for predicting mortality*

Target	AUROC	Precision	Recall	Accuracy	F1 score	Matthews correlation coefficient	Brier score
Training set							
Top 5 features	0.81 (0.75–0.86)	0.59 (0.42–0.76)	0.38 (0.27–0.49)	0.74 (0.67–0.82)	0.44 (0.35–0.53)	0.45 (0.36–0.54)	0.06 (0.05–0.07)
Top 10 features	0.84 (0.78–0.90)	0.40 (0.29–0.52)	0.60 (0.49–0.71)	0.83 (0.77–0.89)	0.46 (0.38–0.54)	0.47 (0.41–0.53)	0.05 (0.04–0.06)
Top 15 features	0.87 (0.82–0.92)	0.49 (0.38–0.60)	0.56 (0.45–0.68)	0.85 (0.81–0.89)	0.49 (0.42–0.56)	0.49 (0.42–0.56)	0.04 (0.04–0.05)
Top 20 features	0.87 (0.82–0.92)	0.45 (0.38–0.53)	0.58 (0.41–0.75)	0.81 (0.75–0.87)	0.47 (0.41–0.53)	0.47 (0.41–0.53)	0.05 (0.04–0.05)
All features	0.84 (0.79–0.89)	0.47 (0.28–0.65)	0.53 (0.44–0.62)	0.82 (0.76–0.89)	0.44 (0.37–0.52)	0.45 (0.37–0.53)	0.05 (0.04–0.05)
Test set							
Top 5 features	0.92	0.30	0.50	0.90	0.38	0.37	0.06
Top 10 features	0.93	0.40	0.67	0.89	0.50	0.50	0.03
Top 15 features	0.97	0.67	0.33	0.94	0.44	0.50	0.02
Top 20 features	0.96	0.36	0.67	0.92	0.47	0.48	0.03
All features	0.96	0.37	0.67	0.91	0.47	0.48	0.03

AUROC: Area under the receiver operating characteristic curve

### SHAP interpreter for the model

**Fig. 2:**
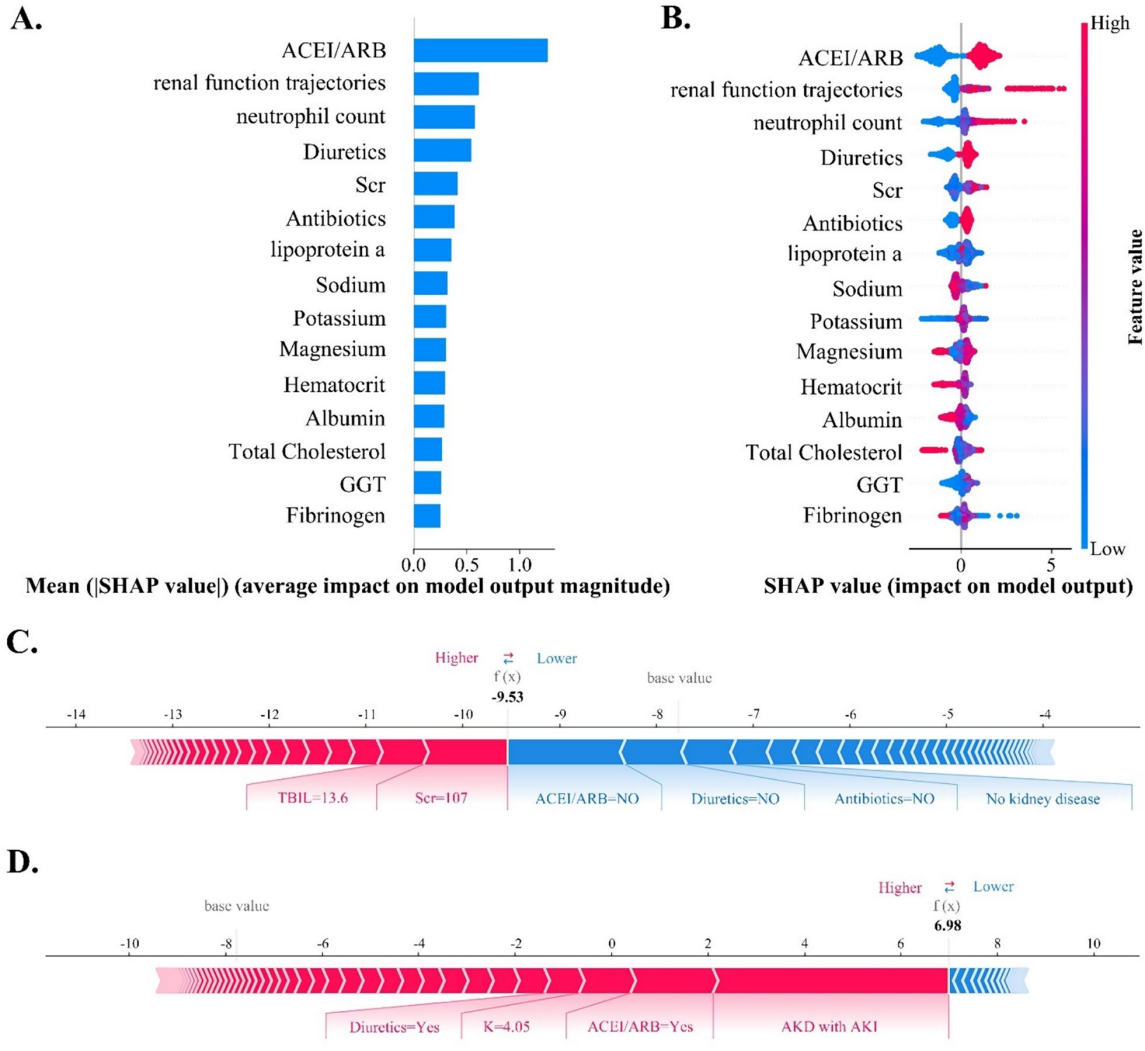
The SHAP summary plots for LightGBM models and force plots for two representative patients. **A** The ranking of feature importance within the mortality prediction model. Features with higher mean absolute SHAP values signify increased predictive influence. **B** Each dot represents the SHAP value of a specific feature for an individual, with red and blue indicating high and low feature values, respectively. On the x-axis, a positive or negative SHAP value signifies that the feature positively or negatively influenced the AKD prediction for the individual. **C** provides a personalized explanation for a case with a mortality probability below 10% and an actual outcome of survival. Features are ranked from the center to both ends based on the extent of their impact. The impact of a feature on the model’s output is directly proportional to the size of the arrow. The positive impact of a feature is depicted in red, elevating the prediction from the base value, while the negative effect is shown in blue, lowering the prediction. Certain features, such as Scr (107 μmol/L) and TBIL (13.6 μmol/L), exhibit a positive influence, while the absence of ACEI/ARB, diuretics, and antibiotics, as well as the absence of kidney disease, contribute negatively to predicting mortality. **D** provides a personalized explanation for a case with a mortality probability exceeding 90% and an actual outcome of mortality. The base value represents the averaged predicted results

Figure 2A, B illustrates the SHAP summary plot of the LightGBM model. The top five features associated with mortality were ACEI/ARE, renal function trajectories (including AKI recovery, subacute AKD, and AKD with AKI), neutrophil count, diuretics use, and Scr. Substituting “AKD grade” for “renal function trajectories” in predicting the risk of mortality resulted in a decrease in the model’s AUROC to 0.92, which was lower than the predictive model constructed by combining AKI and AKD. Furthermore, the importance ranking of “AKD grade” falls outside the top 15 and is not a primary feature for predicting mortality (Fig. S3).

The SHAP interaction plot visually elucidates the interplays among the top 15 features in mortality model (Fig. S4). SHAP dependence plots illustrate the impact of a single feature or the interaction between two features on mortality prediction (Fig. S5). The force plots (Fig. 2C, D) depict the prediction process for two representative patients. The cases shown in Fig. S6 illustrate patients with similar predicted probabilities, yet the constituent feature compositions leading to these predictions differ.

### AI-driven web application

Employing LightGBM for mortality prediction, we have created an AI-driven web application within the Streamlit framework. In the test set (Table 2), compared to the LightGBM model built with all features, the model constructed with the top ten features showed no significant decrease in accuracy (0.89 vs. 0.91) and AUROC (0.93 vs. 0.96), with a slight increase in the F1 score (0.50 vs. 0.47). Therefore, this study utilizes the top ten features for constructing an online predictive model. When users visit the website, they input features data, which is then encoded and sent to the server for real-time mortality prediction. No private data are required besides feature information, and all input is promptly deleted after generating the prediction result. The calculator is accessible at https://strokemortalityapppy-gupkbhhnwkoghqnhvtul8b.streamlit.app/.

## Discussion

To the best of our knowledge, this study is the first to develop and compare multiple ML models for predicting mortality in AIS patients using AKD data. Among 1633 AIS patients, the mortality rate was 4.84%, and 30.31% of patients developed acute/subacute kidney dysfunction. Of these, 65 (3.98%) met both AKI and AKD criteria, 257 (15.74%) developed subacute AKD, and 173 (10.59%) experienced recovery from AKI. LightGBM demonstrated the strongest predictive performance, achieving an AUROC of 0.96 for mortality prediction. The five most important features for assessing mortality risk are ACEI/ARE, renal function trajectories, neutrophil count, diuretic use, and Scr. Compared to using AKD alone, the combined use of AKI and AKD enhances the model’s predictive performance. We further employ various SHAP plots to interpret the “black box model” at both the global and local levels. Ultimately, an AI-driven web application based on the LightGBM model was created for inputting patient data to facilitate the clinicians’ assessment of mortality in AIS patients.

Huang et al. developed various ML algorithms, including eXtreme Gradient Boosting (XGBoost), to develop a mortality prediction model for severe stroke patients [37]. XGBoost outperforms traditional regression models, especially in handling imbalanced and high-dimensional data. Our study compared different ML models using AUROC and F1 scores, and LightGBM demonstrated superior predictive performance. In contrast to XGBoost, LightGBM effectively mitigates overfitting through gradient-based one-side sampling and exclusive feature bundling. In addition, it enhances computational speed and reduces memory usage by employing histogram techniques and a leaf-wise growth strategy [27].

The prediction of mortality risk in AIS patients primarily focuses on ICU patients [22, 23, 37]. Wang et al. developed a mortality prediction model for non-ICU AIS patients using various ML algorithms [24]. However, this study encountered data imbalance issues that remained unaddressed. Several investigations employing regression models have identified AKI and CKD as significant risk factors for mortality in AIS patients [38–40]. The impact of renal function trajectory between 7 and 90 days on mortality remains unclear. This study marks the first attempt to analyze the relationship between AKD and mortality in AIS patients. It underscores that comprehensive renal function trajectories encompassing both AKI and AKD are more vital and precise in predicting mortality risk compared to isolated AKD. This highlights the importance of monitoring the renal function trajectory from 7 to 90 days, even when AIS patients have subacute kidney dysfunction or experience rapid kidney function recovery within 7 days after AKI.

Our study utilized a variety of SHAP plots to address the challenge of the ‘black box’ in mortality risk assessment. Among these, the SHAP summary plot prioritized features based on their importance, identifying ACEI/ARB and renal function trajectories as the two most critical indicators for predicting mortality. SHAP dependence plots demonstrated that patients with acute or subacute kidney injury, particularly those with AKD and AKI, showed an increased risk of mortality associated with ACEI/ARB use. SHAP force plots and decision plots revealed variations in feature contributions for patients with similar predicted probabilities, effectively enhancing the personalization and transparency of the decision-making process.

Our study has some limitations to acknowledge. First, this study lacks specific stroke-related information that could influence mortality, such as the NIHSS score. Second, the follow-up period was too brief to ascertain whether patients developed CKD. Consequently, this study did not assess the influence of AKD on the emergence of new-onset CKD. Third, we have no data specifying the time interval between AIS onset and Scr measurement. However, patients with acute strokes are usually promptly admitted to the hospital, and blood samples are drawn shortly after their arrival. Consequently, the time lapse is unlikely to exceed a few hours. Forth, the AI-driven web application is crafted to assist clinicians in discerning AIS patients with elevated risk of mortality, rather than serving as a replacement for clinical diagnosis. Due to the retrospective nature of data collection, it is crucial to undertake additional validation using an independent population to ensure robust predictive validity across diverse usage scenarios. Fifth, our study is limited to a single center. To enhance the robustness of our findings and ensure their applicability across various scenarios, we will validate our results using an independent population.

## Conclusions

In summary, AKD plays a crucial role in evaluating the mortality risk of AIS patients. Comprehensive renal function trajectories, encompassing both AKI and AKD, are of paramount importance for predicting mortality. The LightGBM model exhibited robust performance as a tool for mortality prediction in AIS patients. The utilization of this AI-driven web application has the potential to significantly reduce mortality rates and assist physicians in making informed treatment decisions.

### Supplementary Information

Additional file 1. Fig. S1. ROC curves and AUROC values for mortality prediction in test set. Fig. S2. ROC (**A**) and DCA (**B**) curves of training and test sets for mortality prediction. Fig. S3. The SHAP summary plot of LightGBM model using “AKD grade” as a proxy for “renal function trajectories” in predicting mortality risk. Fig. S4. The SHAP interaction plot depicting the interactions among the top fifteen features of the lightGBM model for mortality prediction. Fig. S5. The SHAP dependence plots illustrate the correlations between key features in the prediction of mortality. **A** depicts the correlation between renal function trajectories and ACEI/ARB in predicting mortality. The x-axis represents the actual values of renal function trajectories, whereas the y-axis shows the SHAP values for trajectories, with values above zero suggesting an increased risk of mortality. Each dot represents an individual case, with the color transitioning from blue to red to indicate whether ACEI/ARB were taken or not. Specifically, the impact of ACEI/ARB on the mortality probability varies across different renal function trajectories. Among patients with normal kidney function, the use of ACEI/ARB is associated with a decrease in the risk of mortality. Conversely, for patients with AKD accompanied by AKI, the use of ACEI/ARB significantly increases the risk of mortality. **B** Illustrates the correlation between baseline eGFR and ACEI/ARB in predicting mortality. Among patients with lower baseline eGFR levels, the use of ACEI/ARB is associated with a slight increase in the risk of mortality. **C** Depicts the correlation between neutrophil count and renal function trajectories in predicting mortality. **D** Depicts the correlation between neutrophil count and antibiotics in predicting mortality. Fig. S6. The SHAP decision plots provided a detailed view of the inner workings of the lightGBM model. **A**, **B** provides personalized explanations for two cases with mortality probabilities below 10% and actual outcomes of survival. The direction of the line visualizes the decision process of the LightGBM model from the base value to the predicted value. The values adjacent to the line denote the measured values of the features. **C**, **D** provides personalized explanations for two cases with mortality probabilities exceeding 90% and actual outcomes of death. Table S1. Characteristics of hospital encounters in the study sample, overall and according to mortality [mean ± SD; *n* (%)]. Table S2. Performance of eight ML models for predicting mortality. Table S3. Performance of the LightGBM model for predicting mortality in the test set without data balancing.

## Data Availability

The datasets used and/or analyzed during the current study are available from the corresponding author on reasonable request.

## Abbreviations

ACEI/ARB: Angiotensin-converting enzyme inhibitor/angiotensin receptor blocker
ADQI: Acute disease quality initiative
AI: Artificial intelligence
AIS: Acute ischemic stroke
AKD: Acute kidney disease
AKI: Acute kidney injury
AUROC: Area under the receiver operating characteristic curve
BSL: Brier score loss
CKD: Chronic kidney disease
CKD-EPI: The Chronic Kidney Disease Epidemiology Collaboration
DCA: Decision curve analysis
eGFR: Estimated glomerular filtration rate
GBM: Gradient boosting machine
ICU: Intensive care unit
KDIGO: Kidney disease improving global outcomes
KNN: K-nearest neighbors
LightGBM: Light gradient boosting machine
LR: Logistic regression
ML: Machine learning
MLP: Multi-layer perceptron
NB: Naive Bayes
NKD: No kidney disease
RF: Random forest
Scr: Serum creatinine
SD: Standard deviation
SHAP: Shapley additive explanations
SVM: Support vector machine
XGBoost: Extreme gradient boosting
