# Supplementary material for: Personalized prediction of mortality in patients with acute ischemic stroke using explainable artificial intelligence
Source: Eur J Med Res. 2024 Jun 20;29:341. doi: 10.1186/s40001-024-01940-2 (PMC11188208; doi:10.1186/s40001-024-01940-2)
Supplement: Supplementary file 1 — Additional file 1. Fig. S1. ROC curves and AUROC values for mortality prediction in test set. Fig. S2. ROC (A) and DCA (B) curves of training and test sets for mortality prediction. Fig. S3. The SHAP summary plot of LightGBM model using “AKD grade” as a proxy for “renal function trajectories” in predicting mortality risk. Fig. S4. The SHAP interaction plot depicting the interactions among the top fifteen features of the lightGBM model for mortality prediction. Fig. S5. The SHAP dependence plots illustrate the correlations between key features in the prediction of mortality. A depicts the correlation between renal function trajectories and ACEI/ARB in predicting mortality. The x-axis represents the actual values of renal function trajectories, whereas the y-axis shows the SHAP values for trajectories, with values above zero suggesting an increased risk of mortality. Each dot represents an individual case, with the color transitioning from blue to red to indicate whether ACEI/ARB were taken or not. Specifically, the impact of ACEI/ARB on the mortality probability varies across different renal function trajectories. Among patients with normal kidney function, the use of ACEI/ARB is associated with a decrease in the risk of mortality. Conversely, for patients with AKD accompanied by AKI, the use of ACEI/ARB significantly increases the risk of mortality. B Illustrates the correlation between baseline eGFR and ACEI/ARB in predicting mortality. Among patients with lower baseline eGFR levels, the use of ACEI/ARB is associated with a slight increase in the risk of mortality. C Depicts the correlation between neutrophil count and renal function trajectories in predicting mortality. D Depicts the correlation between neutrophil count and antibiotics in predicting mortality. Fig. S6. The SHAP decision plots provided a detailed view of the inner workings of the lightGBM model. A, B provides personalized explanations for two cases with mortality probabilities below 10% and actual [file 40001_2024_1940_MOESM1_ESM.pdf]

Table S1. Characteristics of hospital encounters in the study sample, overall and according to mortality (mean±SD; n (%))*.

Features	Total (n=1633)	Non-mortality (n=1554)	Mortality (n=79)	*P*-value
Demographics				
Age (years)	68.37 ± 13.38	68.13 ± 13.30	73.09 ± 14.13	**<0.01**
Male (%)	991 (60.69)	941 (60.55)	50 (63.29)	0.71
Smokers (%)	559 (34.23)	531 (34.17)	28 (35.44)	0.91
Drinkers (%)	442 (27.07)	419 (26.96)	23 (29.11)	0.77
Surgical history	579 (35.46)	550 (35.39)	29 (36.71)	0.91
Blood transfusion (%)	167 (10.23)	160 (10.30)	7 (8.86)	0.83
BMI (kg/m^2)	23.99 ± 3.22	24.01 ± 3.19	23.65 ± 3.83	0.33
SBP (mmHg)	142.79 ± 23.71	142.68 ± 23.26	144.96 ± 31.44	0.4
DBP (mmHg)	81.34 ± 13.99	81.36 ± 13.80	80.77 ± 17.45	0.71
Fever (%)	137 (8.39)	125 (8.04)	12 (15.19)	**0.04**
Respiratory rate (bpm)				
< 12	3 (0.18)	3 (0.19)	0 (0.00)	0.83
12-20	1371 (83.96)	1306 (84.04)	65 (82.28)	
> 20	259 (15.86)	245 (15.77)	14 (17.72)	
Heart rate (bpm)				
< 60	135 (8.27)	130 (8.37)	5 (6.33)	0.74
60-100	1376 (84.26)	1307 (84.11)	69 (87.34)	
> 100	122 (7.47)	117 (7.53)	5 (6.33)	
Laboratory tests				
RBC (×10^{12}/L)	8.27 ± 4.30	8.09 ± 4.02	11.84 ± 7.12	**<0.01**
WBC (×10^9/L)	4.20 ± 0.76	4.22 ± 0.75	3.90 ± 0.82	**<0.01**
Neutrophil count (×10^9/L)	5.89 ± 3.62	5.70 ± 3.33	9.63 ± 6.22	**<0.01**
Hemoglobin (g/L)	126.30 ± 23.92	126.76 ± 23.72	117.22 ± 26.09	**<0.01**
Platelet (×10^9/L)	217.02 ± 80.16	217.74 ± 80.20	202.90 ± 78.49	0.11
MCV (fL)	89.88 ± 6.19	89.85 ± 6.16	90.52 ± 6.74	0.35
Hematocrit (%)	37.66 ± 6.86	37.76 ± 6.81	35.69 ± 7.66	**0.01**

MCHC (g/L)	334.36 ± 14.96	334.47 ± 15.00	332.29 ± 14.06	0.21
MCH (pg)	30.06 ± 2.43	30.06 ± 2.44	30.05 ± 2.32	0.99
PT (s)	11.44 ± 3.07	11.40 ± 3.04	12.23 ± 3.63	**0.02**
PTA (%)	110.89 ± 32.64	111.33 ± 32.71	102.21 ± 29.95	**0.02**
Fibrinogen (g/L)	3.62 ± 1.10	3.62 ± 1.10	3.81 ± 1.20	0.12
Scr (μmol/L)	102.74 ± 99.40	101.53 ± 98.14	126.59 ± 119.86	**0.03**
Basline eGFR (ml/min/1.73 m^2)	72.43 ± 25.28	72.89 ± 24.99	63.42 ± 29.17	**<0.01**
BUN (mmol/L)	7.01 ± 4.87	7.02 ± 4.91	6.85 ± 3.87	0.77
Uric acid (μmol/L)	289.96 ± 121.17	288.96 ± 119.09	309.59 ± 156.17	0.14
ALT (U/L)	37.52 ± 173.35	36.86 ± 175.32	50.49 ± 128.77	0.5
AST (U/L)	35.22 ± 158.95	33.62 ± 155.23	66.65 ± 218.76	0.07
GGT (U/L)	43.37 ± 83.36	43.04 ± 84.54	49.95 ± 55.19	0.47
ADA (U/L)	12.51 ± 7.18	12.44 ± 7.22	13.91 ± 6.17	0.08
LDH (U/L)	211.61 ± 160.31	207.04 ± 138.41	301.47 ± 384.29	**<0.01**
ALP (U/L)	80.32 ± 54.03	80.22 ± 54.78	82.27 ± 36.62	0.74
Total bilirubin (μmol/L)	17.76 ± 17.93	17.76 ± 18.13	17.79 ± 13.48	0.99
Total protein (g/L)	62.77 ± 7.65	62.85 ± 7.61	61.10 ± 8.23	0.05
Albumin (g/L)	34.71 ± 5.86	34.86 ± 5.82	31.81 ± 5.97	**<0.01**
HDLC (mmol/L)	1.18 ± 0.36	1.18 ± 0.36	1.13 ± 0.39	0.2
LDLC (mmol/L)	2.55 ± 1.04	2.56 ± 1.05	2.39 ± 0.95	0.18
Total cholesterol (mmol/L)	4.48 ± 1.56	4.49 ± 1.57	4.25 ± 1.32	0.18
Triglycerides (mmol/L)	1.38 ± 1.15	1.38 ± 1.16	1.30 ± 0.88	0.53
Lipoprotein a (mg/L)	353.00 ± 380.48	351.99 ± 380.44	372.91 ± 383.30	0.63
Blood glucose (mmol/L)	7.18 ± 3.52	7.10 ± 3.43	8.69 ± 4.71	**<0.01**
Sodium (mmol/L)	140.44 ± 5.02	140.51 ± 4.80	139.10 ± 8.19	**0.02**
Calcium (mmol/L)	2.14 ± 0.18	2.14 ± 0.18	2.15 ± 0.26	0.63
Potassium (mmol/L)	4.03 ± 0.55	4.03 ± 0.56	4.07 ± 0.54	0.57
Magnesium (mmol/L)	0.89 ± 0.12	0.89 ± 0.12	0.89 ± 0.10	0.93
Chloride (mmol/L)	103.32 ± 5.64	103.34 ± 5.68	103.02 ± 4.86	0.63
Phosphorus (mmol/L)	1.04 ± 0.30	1.04 ± 0.30	1.06 ± 0.27	0.56

Anion gap (mmol/L)	12.26 ± 3.26	12.29 ± 3.25	11.59 ± 3.57	0.06
Urinalysis				
pH	6.01 ± 0.60	6.00 ± 0.60	6.15 ± 0.61	**0.04**
Specific gravity	1.02 ± 0.01	1.02 ± 0.01	1.02 ± 0.01	0.18
Protein	501 (30.68)	469 (30.18)	32 (40.51)	0.07
Glucose	935 (57.26)	884 (56.89)	51 (64.56)	0.22
Hematuria	1304 (79.85)	1252 (80.57)	52 (65.82)	**<0.01**
Positive fecal occult blood, n (%)	122 (7.47)	120 (7.72)	2 (2.53)	0.14
Comorbidities, n (%)				
Cerebral hemorrhage	110 (6.74)	103 (6.63)	7 (8.86)	0.59
Epilepsy	31 (1.90)	31 (1.99)	0 (0.00)	0.4
Cerebral aneurysm	37 (2.27)	35 (2.25)	2 (2.53)	1
Shock	16 (0.98)	15 (0.97)	1 (1.27)	1
Diabetes mellitus	565 (34.60)	535 (34.43)	30 (37.97)	0.6
Hypertension	1103 (67.54)	1051 (67.63)	52 (65.82)	0.83
CHD	580 (35.52)	538 (34.62)	42 (53.16)	**<0.01**
Heart failure	271 (16.60)	259 (16.67)	12 (15.19)	0.85
Myocardial infarction	92 (5.63)	84 (5.41)	8 (10.13)	0.13
Cardiacarrhythmia	359 (21.98)	336 (21.62)	23 (29.11)	0.15
Urinary tract infection	50 (3.06)	48 (3.09)	2 (2.53)	1
CKD	115 (7.04)	102 (6.56)	13 (16.46)	**<0.01**
COPD	44 (2.69)	39 (2.51)	5 (6.33)	0.09
Pulmonary infection	415 (25.41)	397 (25.55)	18 (22.78)	0.68
Gastrointestinal bleeding	31 (1.90)	30 (1.93)	1 (1.27)	1
Medications, n (%)				
ACEI/ARB	881 (53.95)	809 (52.06)	72 (91.14)	**<0.01**
CCB	776 (47.52)	743 (47.81)	33 (41.77)	0.35
β-blocker	559 (34.23)	517 (33.27)	42 (53.16)	**<0.01**
Diuretics	1081 (66.20)	1005 (64.67)	76 (96.20)	**<0.01**
Proton pump inhibitors	1073 (65.71)	1005 (64.67)	68 (86.08)	**<0.01**

Statins	1119 (68.52)	1069 (68.79)	50 (63.29)	0.37
Antibiotics	987 (60.44)	915 (58.88)	72 (91.14)	**<0.01**
NSAIDs	204 (12.49)	200 (12.87)	4 (5.06)	0.06
Metformin	232 (14.21)	224 (14.41)	8 (10.13)	0.37
Antithrombotic drugs	1442 (88.30)	1377 (88.61)	65 (82.28)	0.13
Adrenergic drugs	291 (17.82)	277 (17.82)	14 (17.72)	1
Cardiac glycosides	253 (15.49)	236 (15.19)	17 (21.52)	0.17
Outcome				
Renal function trajectories, n (%)				
NKD	1138 (69.69)	1115 (71.75)	23 (29.11)	**<0.01**
Subacute AKD	257 (15.74)	246 (15.83)	11 (13.92)	
AKI recovery	173 (10.59)	153 (9.85)	20 (25.32)	
AKD with AKI	65 (3.98)	40 (2.57)	25 (31.65)	
Renal function grade, n (%)				
0	1138 (69.69)	1115 (71.75)	23 (29.11)	**<0.01**
1	359 (21.98)	326 (20.98)	33 (41.77)	
2	82 (5.02)	74 (4.76)	8 (10.13)	
3	54 (3.31)	39 (2.51)	15 (18.99)	
Length of stay (days)	23.02 ± 12.51	23.19 ± 12.42	19.70 ± 13.78	**0.02**

* SD: Standard deviation; BMI: Body mass index; SBP: Systolic blood pressure; DBP: Diastolic blood pressure; RBC: Red blood cell; WBC: White blood cell; MCV: Mean corpuscular volume; MCHC: Mean corpuscular hemoglobin concentration; MCH: Mean corpuscular hemoglobin; PT: Prothrombin time; PTA: Prothrombin activity; Scr: Serum creatinine; eGFR: Estimated glomerular filtration rate; BUN: Blood urea nitrogen; ALT: Alanine transaminase; AST: Aspartate transaminase; GGT: Gamma-glutamyl transferase; ADA: Adenosine deaminase; LDH: Lactate dehydrogenase; ALP: Alkaline phosphatase; HDLC: High-density lipoprotein cholesterol; LDLC: Low-density lipoprotein cholesterol; CHD: Coronary heart disease; CKD: Chronic kidney disease; COPD: Chronic obstructive pulmonary disease; ACEI/ARB: Angiotensin-converting enzyme inhibitor/Angiotensin receptor blocker; CCB: Calcium channel blocker; NSAIDs: Non-steroidal anti-inflammatory drugs; NKD: No kidney disease; AKI: Acute kidney injury; AKD: Acute kidney disease;

Table S2. Performance of eight ML models for predicting mortality*.

Target	AUROC	Precision	Recall	Accuracy	F1 score	Matthews correlation coefficien	Brier score
**Training set**							
LightGBM	0.84 (0.79-0.89)	0.47 (0.28-0.65)	0.53 (0.44-0.62)	0.82 (0.76-0.89)	0.44 (0.37-0.52)	0.45 (0.37-0.53)	0.05 (0.04-0.05)
GBM	0.76 (0.70-0.82)	0.41 (0.23-0.60)	0.39 (0.23-0.54)	0.73 (0.65-0.81)	0.32 (0.25-0.39)	0.33 (0.26-0.40)	0.05 (0.05-0.05)
RF	0.69 (0.61-0.77)	0.27 (0.14-0.41)	0.45 (0.31-0.60)	0.67 (0.61-0.74)	0.26 (0.17-0.35)	0.24 (0.15-0.34)	0.05 (0.05-0.05)
KNN	0.53 (0.48-0.58)	0.15 (0.08-0.21)	0.46 (0.20-0.73)	0.90 (0.88-0.93)	0.17 (0.12-0.22)	0.11 (0.04-0.17)	0.06 (0.06-0.07)
MLP	0.68 (0.56-0.81)	0.34 (0.12-0.56)	0.55 (0.35-0.75)	0.72 (0.65-0.79)	0.30 (0.20-0.41)	0.33 (0.22-0.44)	0.06 (0.05-0.06)
NB	0.73 (0.66-0.80)	0.32 (0.22-0.42)	0.50 (0.39-0.62)	0.80 (0.73-0.86)	0.36 (0.30-0.42)	0.34 (0.28-0.40)	0.30 (0.26-0.34)
SVM	0.69 (0.60-0.77)	0.36 (0.16-0.56)	0.41 (0.28-0.54)	0.73 (0.64-0.81)	0.31 (0.21-0.40)	0.30 (0.20-0.39)	0.05 (0.05-0.05)
LR	0.76 (0.70-0.83)	0.39 (0.20-0.58)	0.45 (0.30-0.60)	0.72 (0.65-0.79)	0.34 (0.27-0.42)	0.35 (0.26-0.44)	0.17 (0.16-0.18)
**Test set**							
LightGBM	0.96	0.37	0.67	0.91	0.47	0.48	0.03
GBM	0.88	0.22	0.33	0.84	0.27	0.27	0.02
RF	0.76	0.08	0.33	0.66	0.13	0.16	0.03
KNN	0.58	0.06	0.33	0.85	0.10	0.09	0.04
MLP	0.46	0.20	0.33	0.95	0.25	0.23	0.03
NB	0.78	0.18	0.67	0.87	0.29	0.32	0.24
SVM	0.70	0.33	0.33	0.61	0.33	0.32	0.03
LR	0.75	0.29	0.33	0.68	0.31	0.29	0.15

* ML: Machine learing; AUROC: Area under the receiver operating characteristic curve; LightGBM: Light Gradient Boosting Machine; RF: Random Forest; KNN: K-Nearest Neighbors; MLP: Multi-Layer Perceptron; NB: Naive Bayes ; SVM: Support Vector Machine; LR: Logistic Regression.

Table S3. Performance of the LightGBM model for predicting mortality in the test set without data balancing*.

Target	AUROC	Precision	Recall	Accuracy	F1 score	Matthews correlation coefficien	Brier score
Top 5 features	0.92	0.22	0.67	0.90	0.33	0.36	0.03
Top 10 features	0.93	0.38	0.50	0.94	0.43	0.45	0.02
Top 15 features	0.96	0.67	0.33	0.92	0.44	0.47	0.02
Top 20 features	0.96	0.32	1.00	0.95	0.48	0.55	0.02
All features	0.92	0.33	0.50	0.89	0.40	0.39	0.03

* AUROC: Area under the receiver operating characteristic curve.

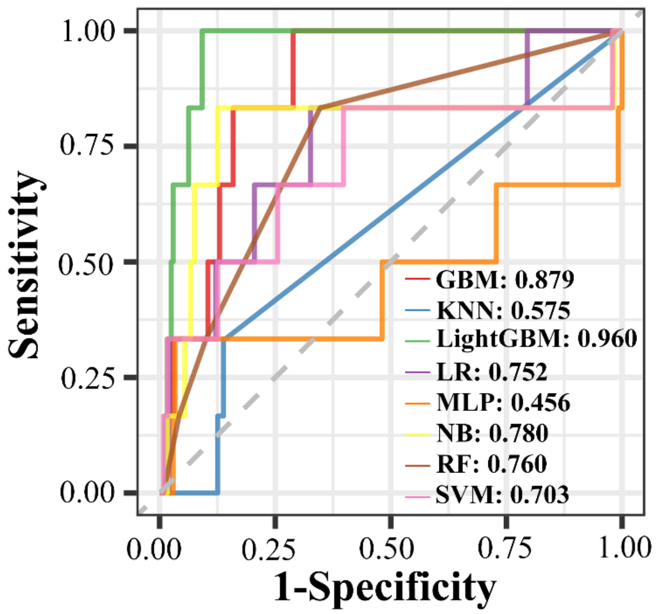

Fig. S1 ROC curves and AUROC values for mortality prediction in test set.

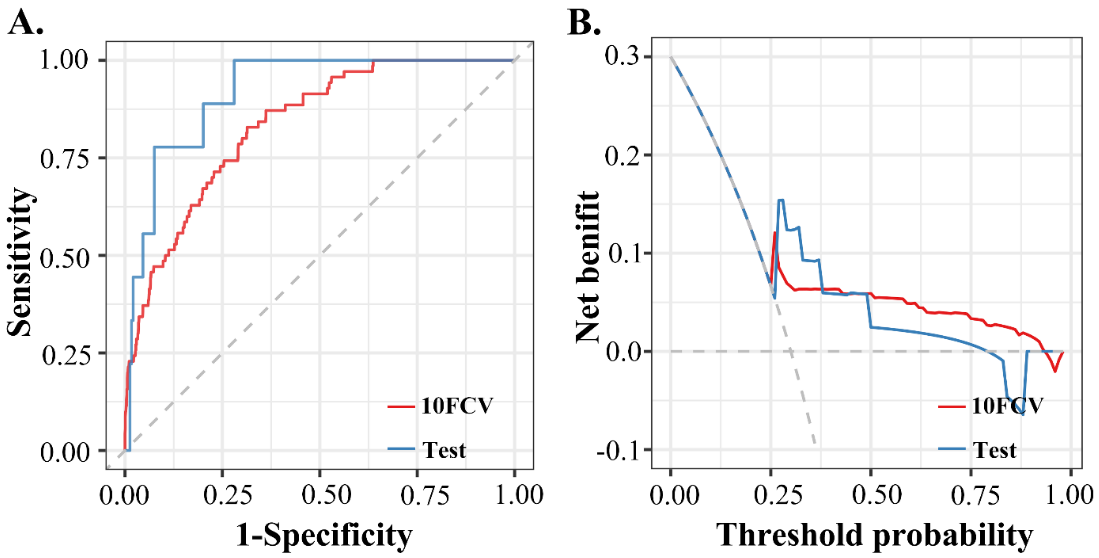

Fig. S2 ROC (A) and DCA (B) curves of training and test sets for mortality prediction.

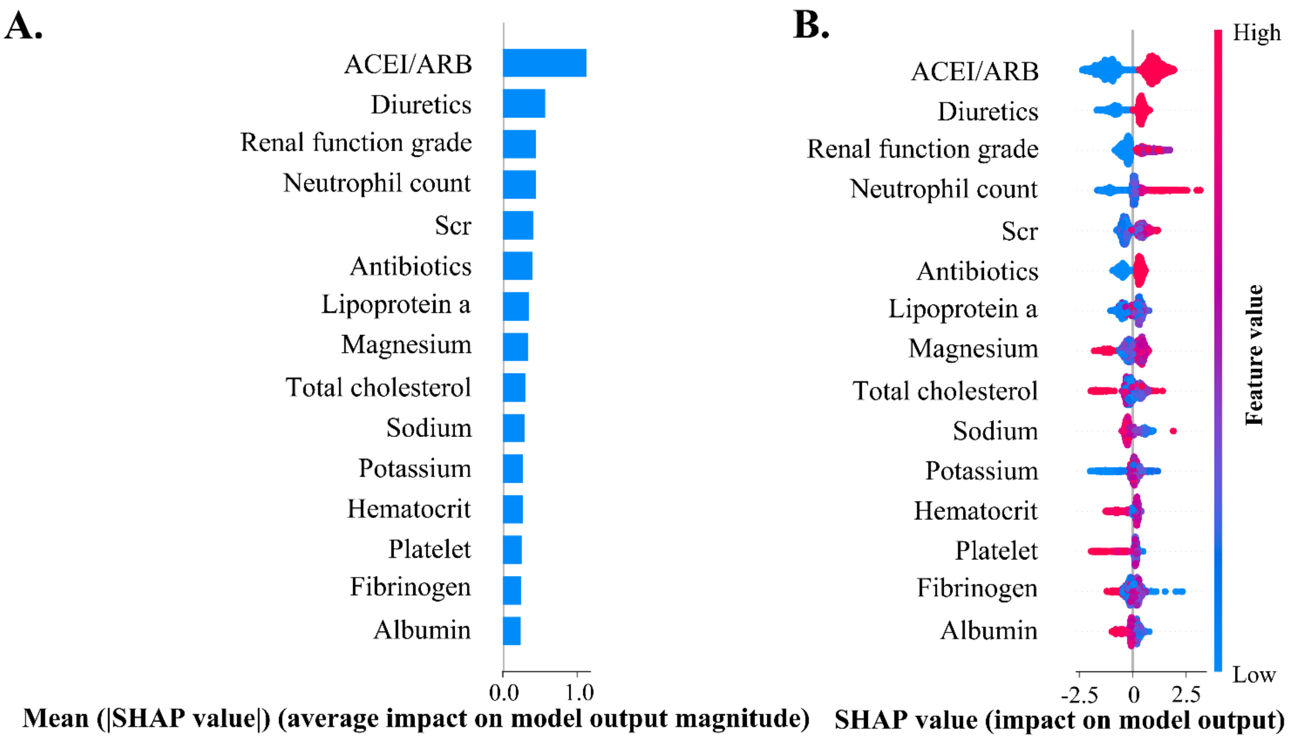

Fig. S3 The SHAP summary plot of LightGBM model using "AKD grade" as a proxy for "renal function trajectories" in predicting mortality risk.

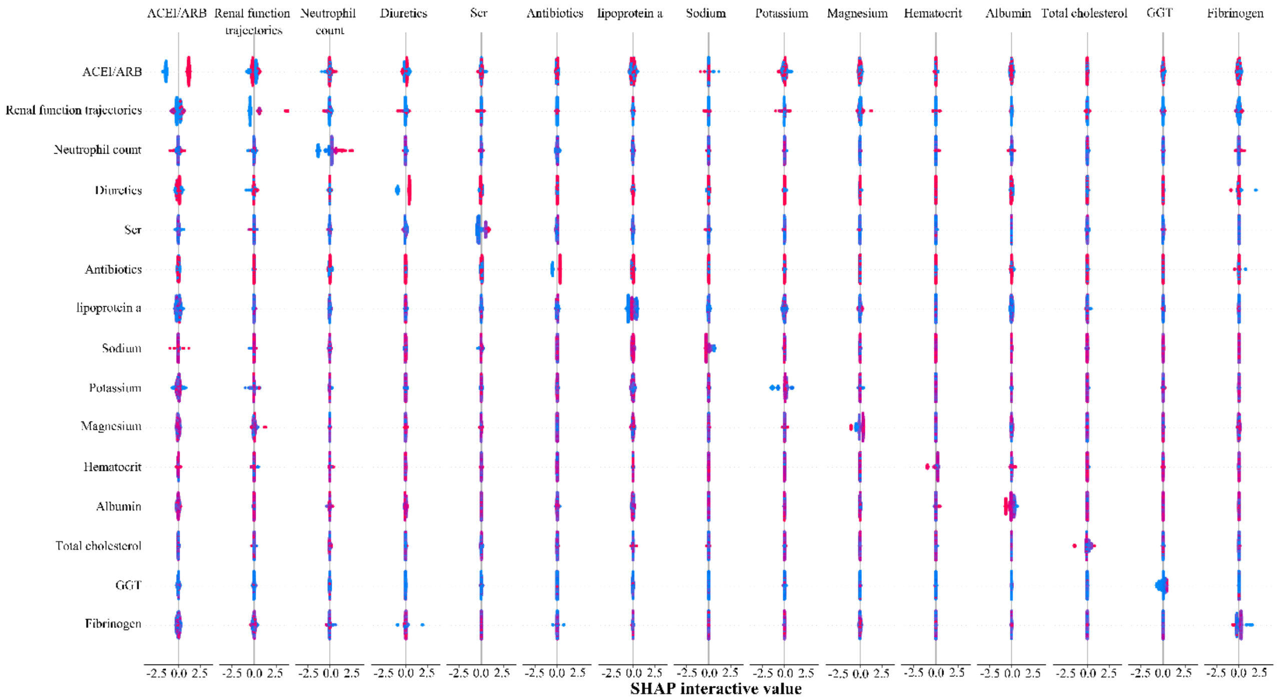

Fig. S4 The SHAP interaction plot depicting the interactions among the top fifteen features of the lightGBM model for mortality prediction.

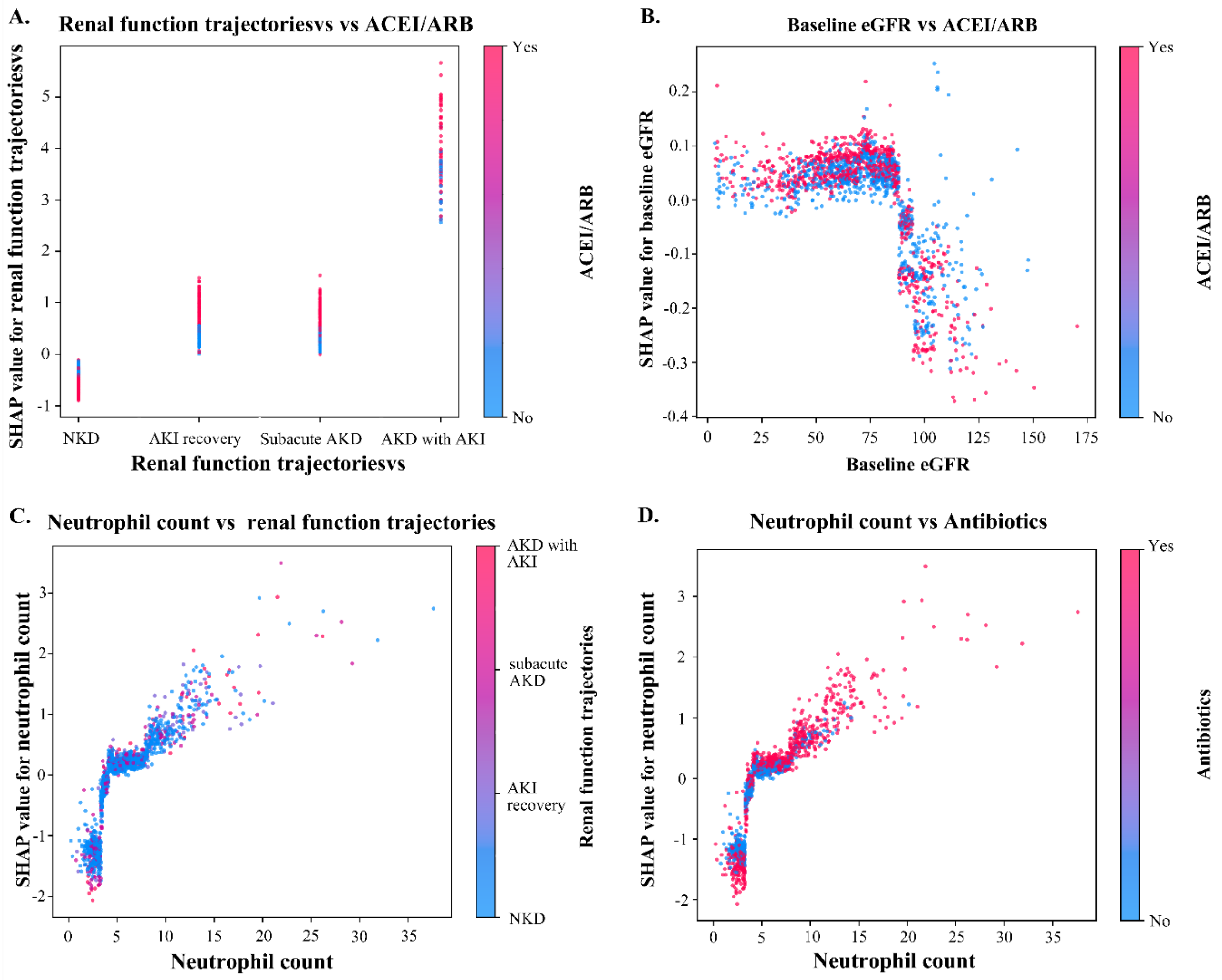

Fig. S5 The SHAP dependence plots illustrate the correlations between key features in the prediction of mortality. **A.** depicts the correlation between renal function trajectories and ACEI/ARB in predicting mortality. The x-axis represents the actual values of renal function trajectories, whereas the y-axis shows the SHAP values for trajectories, with values above zero suggesting an increased risk of mortality. Each dot represents an individual case, with the color transitioning from blue to red to indicate whether ACEI/ARB were taken or not. Specifically, the impact of ACEI/ARB on the mortality probability varies across different renal function trajectories. Among patients with normal kidney function, the use of ACEI/ARB is associated with a decrease in the risk of mortality. Conversely, for patients with AKD accompanied by AKI, the use of ACEI/ARB significantly

increases the risk of mortality. **B.** illustrates the correlation between baseline eGFR and ACEI/ARB in predicting mortality. Among patients with lower baseline eGFR levels, the use of ACEI/ARB is associated with a slight increase in the risk of mortality. **C.** depicts the correlation between neutrophil count and renal function trajectories in predicting mortality. **D.** depicts the correlation between neutrophil count and antibiotics in predicting mortality.

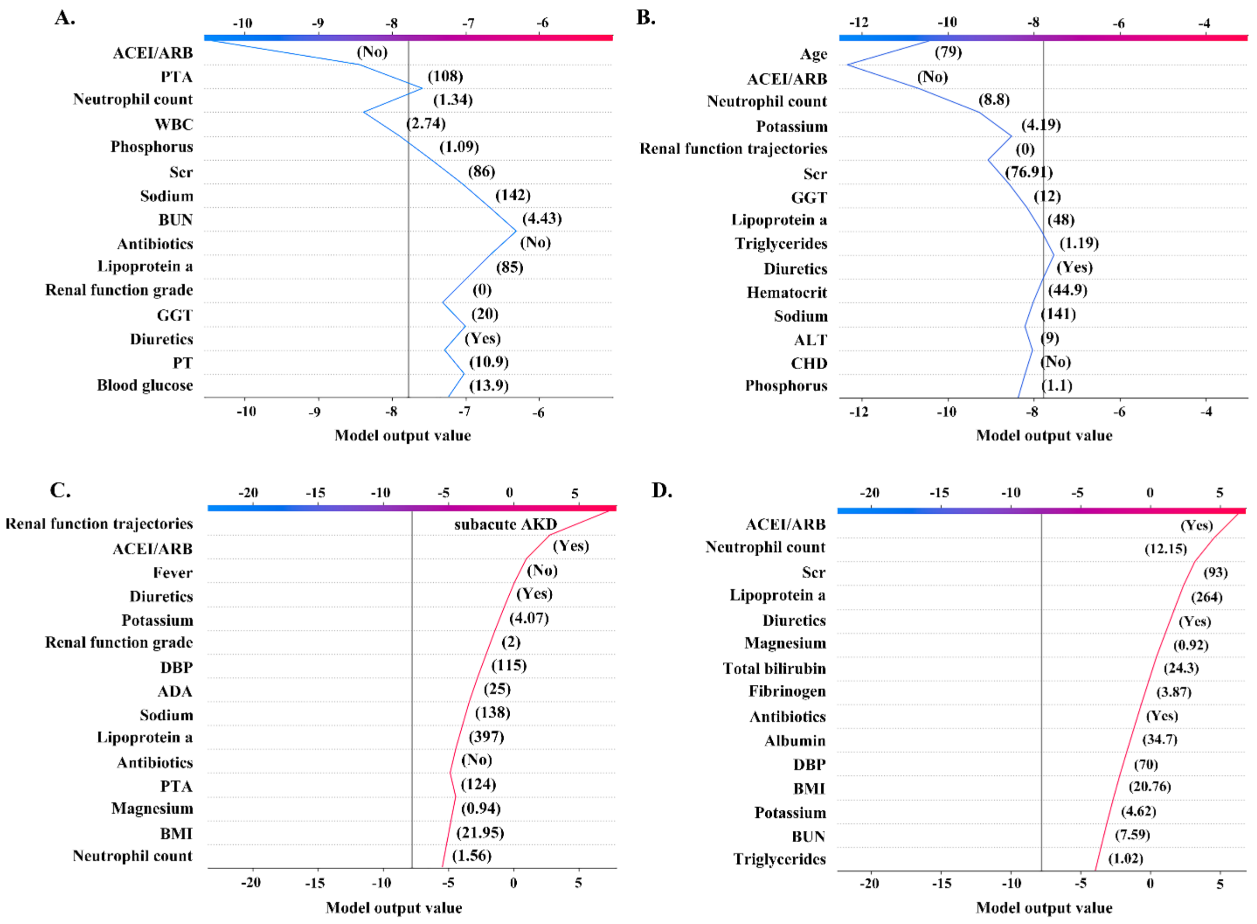

Fig. S6 The SHAP decision plots provided a detailed view of the inner workings of the lightGBM model. **A-B.** provides personalized explanations for two cases with mortality probabilities below 10% and actual outcomes of survival. The direction of the line visualizes the decision process of the LightGBM model from the base value to the predicted value. The values adjacent to the line denote the measured values of the features. **C-D.** provides personalized explanations for two cases with mortality probabilities exceeding 90% and actual outcomes of death.
